# Physicochemical Properties and Biological Activities of Garlic (*Allium sativum* L.) Bulb and Leek (*Allium ampeloprasum* L. var*. Porrum*) Leaf Oil Extracts

**DOI:** 10.1155/2022/6573754

**Published:** 2022-04-26

**Authors:** Elias Lemma, Zekeria Yusuf, Mulugeta Desta, Sultan Seyida, Megersa Idris, Sewnet Mengistu, Jemal Teneshu

**Affiliations:** School of Biological Sciences and Biotechnology, Haramaya University, Dire Dawa, Ethiopia

## Abstract

*Allium* species including garlic and leek exhibits a broad range of medicinal and nutritional properties. Therefore, this study investigates the physicochemical and biological activities of garlic (*Allium sativum* L.) and leek (*A. ampeloprasum* L. var. *Porrum*) oil extracts. The result indicated that physicochemical properties indicated that significantly higher oil yield (21.25%), ACV (2.66 mg/g), FFA (1.34%), and PV (4.10 meq/kg) and also antioxidant activities with respect to 2, 2-diphenyl-1-picrylhydrazyl, DPPH (27.60 ± 1.55%), hydrogen peroxide (12.35 ± 0.92%) free radical scavenging activities, and ascorbic acid content (25.30 ± 3.25%) were obtained for leek leaf oil extract. Stronger antibacterial activity with a maximum zone of inhibition (16.00 mm), minimum inhibitory concentration (MIC) (0.20 *µ*g/ml), and minimum bactericidal concentration (MBC) (0.40 *µ*g/ml) was recorded for leek oil extract against *S. pyogenes*. However, garlic oil has presented stronger antifungal activity with a maximum zone of inhibition (13.50 mm), MIC (0.40 *µ*g/ml), and minimum fungicidal concentration (MFC) (0.75 *µ*g/ml) against *Candida albicans.* It is concluded from the results of this investigation that oils extracts of garlic bulb and leek leaves demonstrated significant biological activities that can be used as sources for pharmaceutical and nutraceutical ingredients.

## 1. Introduction

*Allium* spp. including garlic (*Allium sativum* L.) and leek (*Allium ampeloprasum* L.) [1] exhibits a broad spectrum of pharmacological activities. The medicinal properties of garlic are mainly due to sulfur-containing compounds such as allicin, alliin and ajoene, enzymes, soluble sugars, minerals (germanium, selenium, and zinc), amino acids (cysteine, glutamine, isoleucine, and methionine), flavonoids (quercetin, cyanidin, and allistatins I and II), vitamins (C, E, A, B1, and B2), and *β*-carotene [2]. Cooked and waited garlic extracts and oils can provide better protection against free radicals and infections than fresh garlic [3].

*Allium* spp., such as garlic, onion, and leek, have long been known to be effective in the therapy of infectious diseases. In particular, garlic has a greater antimicrobial activity than other *Allium* spp. as it contains several hydrophobic antimicrobial compounds, such as allicin, vinyldithiin, ajoene, and diallyl polysulfide [4]. Allicin is a characteristic sulfur-containing compound found in raw garlic and exhibits antimicrobial activity against both Gram-positive and Gram-negative bacteria. In addition, allicin has been reported to inhibit the biofilm formation of bacteria, which is a major cause of bacterial resistance to the antibiotic treatment of infections, by regulating quorum sensing in microorganisms [4, 5].

The spread of drug-resistant bacteria has become a serious global concern in the therapy of infectious diseases. A number of antibiotics have been developed and used to treat infectious diseases. However, the increased frequency in the use of such antibiotics has led to changes in bacterial characteristics, with bacteria acquiring drug-resistant ability through the mutations of drug-target molecules, the overexpression of efflux pumps, changes in the composition of the cell membrane, the production of metabolizing enzymes, and biofilm formation [6]. Garlic (*Allium sativum* L.) has been used as not only a food but also as a remedy for several diseases, such as cardiovascular diseases and cancer [7, 8].

Garlic and leek oils are applicable in pharmaceuticals and nutraceutical industries as flavouring agents and can reduce blood pressure and prevent cancer and cardiovascular diseases through reducing serum LDL cholesterol and triglycerides [9]. There are many reports on the antimicrobial, antioxidant, antithrombotic, and anticancer properties of garlic oil, which could be used to prevent nausea, diarrhea, ease coughs, and treatment in conditions such as malaria and cholera [10]. The organosulphur compounds in garlic and onion are responsible for their characteristic odour and flavour, as well as most of their biological activities [11]. The aim of the present study was to assess the physicochemical properties, antioxidant, and antimicrobial activities of oil extracts from garlic bulb and leek leaves.

## 2. Materials and Methods

### 2.1. Plant Material and Extract Preparation

The experiment was conducted in the Molecular Biology and Biotechnology Laboratory, Haramaya University. The garlic (*Allium sativum* L.) bulb and leek (*Allium ampeloprasum* L. var. *Porrum*) leaf samples were collected from a farmland in Kombolcha district, East Hararghe, Ethiopia. The garlic bulb and leek leaf samples were manually washed with distilled water and residual moisture evaporated at room temperature. Determination of moisture (on dry basis) was carried out [12]. The oil extraction was done in the Soxhlet apparatus using hexane as a solvent.

### 2.2. Physicochemical Properties of the Oil Extracts

The oil yield and specific gravity were done based on Soxhlet oil method [13–15]. The percentage oil yield of each sample was determined as follows:

(1) $$\begin{array}{c} \text{Oil yield}=\frac{\text{oil weight}\left(\text{OW}\right)}{\text{sample weight}\left(\text{SW}\right)}x100, \end{array}$$

where oil weight = W2-W1, W1 is the weight of the extraction flask (g), and W2 is the weight of the extraction flask plus the dried crude fat (g).

The specific gravity of oil was determined gravimetrically by employing the weight ratio of oil to the equivalent amount of water according to the following formula:

(2) $$\begin{array}{c} \text{Specific gravity}=\frac{\text{W}2}{\text{W}1}\text{,} \end{array}$$

where W2 and W1 are the weights of oil and equivalent amount of water, respectively.

### 2.3. Determination of Acid Value

The acid value was determined as per the AOAC [16] method. Briefly, 2 g of oil sample was weighed into a 250 ml conical flask, and then, 25 ml diethyl ether mixed with 25 ml alcohol and 1 ml of 1% phenolphthalein indicator were added to the oil sample. The conical flask was then placed on a hot waterbath until oil was completely dissolved in the solvent. The hot solution was then titrated with 0.1 M KOH until a pink colour, which persisted for 15 seconds, was noticed. The acid value was calculated as

(3) $$\begin{array}{c} \text{Acid value}=\frac{\text{Titre}\left(ml\right)x\;5.61}{\text{Weight of sample used}}, \end{array}$$

which is expressed as mg KOH/g of oil.

### 2.4. Estimation of Free Fatty Acid

The percentage of free fatty acid (%FFA) was estimated by multiplying the acid value with the factor 0.503. %FFA = 0.503 × acid value.

### 2.5. Determination of Peroxide Value

To a weighed sample (1.0 g) in a flask was added powdered potassium iodide (1.0 g) and solvent mixture (2 : 1, glacial acetic acid: chloroform v/v). The resulting solution was then placed on a waterbath to dissolve properly, and 5% potassium iodide (20 cm^3^) was then added. The sample solution was then titrated with 0.002 N sodium thiosulphate using starch as an indicator. The peroxide values of the samples were calculated using the following equation [17].

(4) $$\begin{array}{c} \text{PV}=2\text{XV,} \end{array}$$

where PV is the peroxide value, V is the volume of sodium thiosulphate used, 2 = (N x 1000)/W, N is the normality of sodium thiosulphate used, and W is the weight of sample used.

### 2.6. Antioxidant Activity

#### 2.6.1. DPPH Radical Scavenging Activity

The radical scavenging activity (RSA) of the oil extract is used to measure antioxidant activity using the DPPH (2, 2-diphenyl-1-picrylhydrazyl) method [18]. Briefly, 2 ml of oil extract (100 *µ*g/ml) was added to 2 mL of DPPH (0.1 mM) solution. The mixtures were kept aside in a dark area for 30 min and absorbance was measured at *λ*max 517 nm against an equivalent amount of DPPH and methanol as a blank. The percentage of DPPH radical scavenging activity (RSA %) was estimated using the following equation:

(5) $$\begin{array}{c} \text{DPPH radical scanveging activity}\left(\%\right)=\frac{\left(A_{\text{blank}}-A_{\text{sample}}\right)}{A_{\text{blank}}}x100, \end{array}$$

where A_blank_ is the absorbance of the blank solution and A sample is the absorbance in the presence of the sample. Ascorbic acid was used as the positive control.

#### 2.6.2. Hydrogen Peroxide Scavenging Activity

The radical scavenging activity of the oil extract was determined using the H_2_O_2_ method [19]. Briefly, 2 ml of oil extract solution (100 *µ*g/ml) was added to 4.0 ml of H_2_O_2_ (20 mM) solution in phosphate buffer (pH 7.4). After 10 min, the absorbance was measured at *λ*_max_ 230 nm against the phosphate buffer blank solution. The percentage scavenging of H_2_O_2_ was calculated using the equation:

(6) $$\begin{array}{c} \%\text{ scavenging of }{\text{H}}_{2}{\text{O}}_{2}=\;\frac{\left(A_{\text{blank}}-A_{\text{sample}}\right)}{A_{\text{blank}}}x100, \end{array}$$

where A_blank_ is the absorbance of the blank (phosphate buffer with H_2_O_2_) and A_sample_ is the absorbance of the oil extracts.

#### 2.6.3. Ascorbic Acid Content

The ascorbic acid content was determined using the 2, 6-dichlorophenol indophenol (DCPIP) dye method [16]. Accordingly, 5 ml of the standard ascorbic acid solution was pipetted into a 100 ml conical flask, and then, 5 ml of the 3% HPO_3_ solution was added. The ascorbic acid solution was titrated with the dye solution to a pink colour that persisted for 15 s. The titer value was recorded. Dye factor was expressed as mg of ascorbic acid per ml of the dye. Since 5 ml of the standard ascorbic acid solution contains 0.5 mg ascorbic acid,

(7) $$\begin{array}{c} \text{Dye factor }\left(\text{mg ascorbic acid per dye}\right)=\frac{0.5mg}{\text{titrant volume}}. \end{array}$$

One ml of the extracted oil was diluted to 5 ml with 3% metaphosphoric acid in a 50 ml volumetric flask. The aliquot was then centrifuged (Model, Z300, 580W, 3052 Nm, Germany) for 15 minutes and titrated with the standard dye to a pink end point (persisting for 15 seconds). The ascorbic acid content was calculated from the titration value, dye factor, dilution, and volume of the sample as

(8) $$\begin{array}{c} \%\text{A.A}=\frac{\left(\text{ABR sample}\right)\text{x dye factor x volume of initial test solution}}{\text{volume of test solution titrated}}\;x\;100\%, \end{array}$$

where A.A is the ascorbic acid and ABR is the average burette reading.

### 2.7. Antimicrobial Activity of the Oil Extracts

The antimicrobial experiment was arranged as 2X1X4 (2 source extracts: garlic bulb and leek leaves at three concentration levels, 1 solvent system, i.e., hexane, 4 test organisms including two bacteria (*Shigella boyelli* (Gram-negative) and *Streptococcus pyogenes* (Gram-positive)) and two fungi (*Aspergillus niger* and *Candida albicans*)) completely randomized factorial design in three replications. The test pathogens were obtained from Ethiopian Institute of Public Health and Nutrition, Addis Ababa, Ethiopia. The fungal and bacterial pathogens were subcultured and maintained on potato dextrose agar (PDA) and nutrient agar, respectively. Thenceforth, the fungal and bacterial cultures were incubated for 72 h at 27°C and for 24 h at 37°C, respectively.

#### 2.7.1. Media Preparation and Standardization of Inoculum

Nutrient agar (NA), potato dextrose agar (PDA), and Mueller–Hinton agar (MHA) were used for subculturing of bacterial test organism and fungal test organism and determination of antimicrobial activities, respectively. These media were prepared and sterilized using an autoclave according to the manufacturers' instructions. Two to three bacterial colonies on the plate were picked up with a sterile inoculating loop and transferred into a test tube containing sterile normal saline and vortexes thoroughly. The spores of the test fungi were harvested by washing the surface of the fungal colony using 5 ml of sterile saline solution. This procedure was repeated until the turbidity of each bacterial and fungal spore suspension matched the turbidity of 0.5 McFarland Standards as described by the Clinical Laboratory Standards Institute [20]. The resulting suspension was used as inoculums for the test pathogen in the antimicrobial susceptibility test.

#### 2.7.2. Disc Diffusion Method

The discs of 6 mm diameter were prepared from sterile filter paper cut into small, circular pieces of equal size by a perforator and then impregnated. Each of them was impregnated with 0.01 ml of the prepared test extract ethyl acetate solution. The extract impregnated discs were placed onto MHA plates evenly inoculated with test pathogens [21]. Following this step, the impregnated discs were dispensed onto the surface of the inoculated agar plates using sterile forceps [22]. Discs of commercial ampicillin (1 *µ*g/disc) and ketoconazole (1 *µ*g/disc) were used as positive controls for bacterial and fungal pathogens, respectively, and distilled water impregnated discs were used as negative controls. Then, the MHA plates were sealed with parafilm and incubated at 37°C for 24 h and 27°C for 72 h for bacterial and fungal pathogens, respectively. The diameters of the zone of inhibition around each disc were measured to the nearest millimeter along two axes (i.e., 90° to each other) using a transparent ruler, and the means of the two readings were recorded.

#### 2.7.3. Determination of Minimum Inhibitory Concentration (MIC)

The oil extracts that showed significant antimicrobial activity in the antimicrobial activity tests were selected for determination of MIC based on the broth dilution method followed by Mousaviet al.[23] with slight modifications. In the broth dilution method, two milliliter of nutrient broth and potato dextrose broth for bacteria and fungi, respectively, were added into all test tubes and 0.1 ml of the prepared concentration of each oil extract was mixed with the nutrient broth and potato dextrose. Thereafter, standardized inoculums of 0.1 ml of the respective test pathogens were dispensed into the test tubes containing the suspensions of the broth and the oil extract. Then, all test tubes were properly corked and incubated at 37°C for 24 h for bacteria and 27°C for 72 h for fungi. After that, they were observed for the absence or presence of visible growth. The lowest concentration at which no visible growth of organisms occurs was regarded as the MIC. The experiment was carried out for each test organism in triplicates.

#### 2.7.4. Determination of Minimum Bactericidal (MBC) and Fungicidal Concentrations (MFC)

For the determination of the MBC and MFC, fresh nutrient agar and potato dextrose agar plates were inoculated with a loop taken from each of the broth cultures that showed no growth in the MIC tubes, that is, MBC/MFC values were determined by subculturing from respective MIC values. Antibacterial agents are usually regarded as bactericidal if the MBC is no more than four times the MIC [20]. MBC/MFC is the amount of the extract that kills microbial growth. While MBC assay plates were incubated for 48 h, MFC assay plates were incubated for 3 days. After the incubation periods, the lowest concentration of the extract that did not allow any bacterial or fungal growth on solid medium was regarded as MBC and MFC for the extract [22].

### 2.8. Statistical Data Analysis

The experimental data (Supplementary Tables S1, S2, and S3) were subjected to analysis of variance (ANOVA) using ProcGLM and least significance difference (LSD) *t*-test programs of SAS version 9.2 [24] to investigate the statistical significance between different oil quality parameters. Differences between means were considered statistically significant at *p* < 0.05.

## 3. Result and Discussion

### 3.1. Physicochemical Properties of Oils from Garlic (*Allium sativum* L.) Bulbs and Leek (*Allium ampeloprasum* L.) Leaf Oils

Physicochemical properties of *A. sativum* bulb and *A. ampeloprasum* leaf oil extracts were determined based on oil content, specific gravity, acid value, free fatty acids, and peroxide values as given in Table 1. Significance differences between garlic bulbs and leek leaf oils were obtained for all measured parameters except for specific gravity. Significantly higher oil yield (21.25%), ACV (2.66 mg/g), FFA (1.34), and PV (4.10 meq/kg) were recorded for leek leaf oil extract. A similar study was conducted by Rafe and Nadjafi [25] in which the chemical analyses revealed 2.5 as the acid value, 1.27 for free fatty acids, 1.8 as the saponification value, and 14.5 as the iodine value of garlic oil.

Shalaby et al. [26] indicated volatile oil composition of (0.073% ± 0.1) for *A. sativum* and (0.59% ± 0.0) for *A. cepa.* Boukeria et al. [27] suggested that variation observed in essential oils yield, between varieties of each species, may be explained by the differences in genetic background of the targeted plant and/or to the extraction protocol followed during the experiment. Specific gravity is a characteristic used in the classification of essential oils. The specific gravity of oil is defined as the mass ratio of a volume of oil to that of an equal volume of distilled water, both held at 20°C [28]. Measuring the specific gravity of different essential oils showed a higher density than water, allowing them to be below water during extraction. The acid index gives an idea about the content of free acids. The acid number less than 2 indicates proper preservation of these oil's weak quantity of free acid [29].

### 3.2. Antioxidant Activities of Garlic Bulb and Leek Leaf Oil Extracts

The antioxidant activities of the oil extracted from garlic and onion bulbs were assessed based on DPPH and hydrogen peroxide free radical scavenging activities and ascorbic acid content as given in Table 2. Significantly higher antioxidant activities with respect to DPPH (27.60 ± 1.55%), hydrogen peroxide (12.35 ± 0.92%) free radical scavenging activities, and ascorbic acid content (25.30 ± 3.25%) were obtained for leek leaf oil extract. The higher DPPH value (27.60%) indicates higher antioxidant activities and the presence of higher essential omega-3 fatty acids in leek leaf oil extract. The antioxidant activities of leek leaf oil were found to be significantly higher indicating that leek oil might possess better biological activities, oil quality, and pharmacological applications. A similar study was conducted by Abdel-Salam et al. [30] who suggested the phenolic contents and antioxidant activity were higher in essential oils of garlic, leek, and red onion, whereas the lower contents and activity were shown in aqueous extracts.

The DPPH and oxygen radical absorption capacity (ORAC) assays showed that the ethanolic extract of garlic sprouts exhibited stronger antioxidant activities than the ethanolic extract of raw garlic [31]. In addition, the antioxidant properties of aged garlic were found to be higher than fresh garlic by DPPH, ABTS, FRAP, H_2_O_2_ scavenging, and Fe2 + chelating assays [32]. Compared with multi clove garlic extract, single clove garlic extract had a higher amount of phenolic compounds and showed stronger antioxidant activity [33]. Moreover, the antioxidant activity of black garlic increased with thermal treatment, and the highest antioxidant activity was obtained on the 21^st^ day of processing [34, 35]. Also, the increased pressure improved the antioxidant activity of garlic paste [36]. However, the antioxidant activity of “Laba” garlic, a traditional Chinese garlic product, decreased during fermentation [37].

### 3.3. Antimicrobial Activities of Garlic Bulb and Leek Leaf Oil Extracts

The disk diffusion method was used to measure the diameter of inhibition zone. The diameter of inhibition zone for garlic and leek leaf oil extracts is given in Table 3. Both garlic and leek oil extracts presented significantly lower inhibition as compared to positive controls including ampicillin for the antibacterial activity test and ketoconazole for the antifungal activity test. Significance differences were recorded for both garlic and onion bulb oil extracts at different concentration levels. The mean zone of inhibition at highest concentration (3 *µ*g/ml) against bacterial test pathogens ranged from 12.93 ± 0.40 mm to 16.00 ± 0.50 mm, while 0.00 ± 0.00 to 13.50 ± 0.45 mm against fungal test pathogens. Stronger antibacterial activity with maximum zone of inhibition (16.00 mm) was recorded for leek oil extract against *S. pyogenes* indicating that *S. pyogenes* was more susceptible than *S. boyelli.*

On the other hand, the stronger antifungal activity with maximum zone of inhibition (13.50 mm) was recorded for garlic bulb oil extract against *Candida albicans* as the weaker antifungal activity with zero zone of inhibition (0.00 mm) was observed for leek oil against *A. niger.* The crude garlic oil extracted by diethyl ether solvent exhibited marked inhibition activity against bacteria, and inhibition was stronger than those of garlic oil extracted by hydrodistillation [38]. *Allium* essential oils were more effective as compared to aqueous extracts. At 100% dilution, the maximum zone of inhibition was observed against *K. oxytoca*, a Gram-negative organism, and the minimum was against *E. coli*, a Gram-negative, and *S. aureus*, a Gram-positive organism, for the garlic essential oil [39].

### 3.4. Minimum Inhibitory Concentration (MIC), Minimum Bactericidal Concentration (MBC), and Minimum Fungicidal Concentration (MFC) of Oils from Garlic Bulb and Leek Leaf Oil Extracts

The antimicrobial efficacy of garlic bulb and leek leaf oil extracts against pathogenic microbes was evaluated by MIC, MBC, and MFC as given in Table 4. The oil extracts from leek leaf have exhibited stronger bactericidal activity with MIC (0.20 *µ*g/ml) and MBC (0.45 *µ*g/ml) against *S. pyogenes* indicating that *S. pyogenes* was more susceptible to the oil extract and also indicating leek oil possesses stronger antibacterial potential. By contrast, garlic bulb oil extract has demonstrated stronger antifungal activity with MIC (0.40 *µ*g/ml, the least value) and MFC (0.75 *µ*g/ml) against *C. albicans* showing that *C. albicans* was more susceptible to the oil extract than *A. niger*, and the garlic bulb oil was more effective antifungal potential than leek leaf oil extract.

## 4. Conclusion

The result indicated that oils extracts of both garlic bulb and leek leaves demonstrated considerable biological activities as antioxidant and antimicrobial potentials. Leek oil was found to have stronger antioxidant and antibacterial potential. However, garlic bulb oil demonstrated better antifungal potential. Both garlic and leek oil extracts exhibited marked inhibition activity against bacteria at high concentrations. Therefore, the oil extracts of both *Allium* spp. can be used as a potential source of ingredients for sustainable pharmaceutical and nutraceutical industries in further study conducted on them.

## Figures and Tables

**Table 1:** Physicochemical properties of garlic bulb and leek leaf oils.

Oil extract	Oil yield (g/g)	Specific gravity	ACV (mg/g)	FFA (%)	PV (meq/kg)
Leek	21.25 ± 1.80^a^	0.85 ± 0.07^a^	2.66 ± 0.20^a^	1.34 ± 0.10^a^	4.10 ± 0.12^a^
Garlic	17.50 ± 2.12^b^	0.70 ± 0.08^a^	1.54 ± 0.30^b^	0.70 ± 0.01^b^	2.20 ± 0.14^b^

Means followed by same letter within a column were not significantly different at 0.05 probability level based on the LSD (least significance difference) test. ACV, acid value; FFA, free fatty acids; PV, peroxide value.

**Table 2:** Antioxidant activities of garlic bulbs and leek leaf oils.

Oil extract	DPPH	HPSA	Ascorbic acid
Leek	27.60 ± 1.55^a^	12.35 ± 0.92^a^	25.30 ± 3.25^a^
Garlic	17.25 ± 1.20^b^	8.95 ± 1.50^b^	20.77 ± 3.26^b^

Means followed by same letter within a column were not significantly different at 0.05 probability level based on the LSD (least significant difference) test. DPPH, 2, 2-diphenyl-1-picrylhydrazyl; HPSA, hydrogen peroxide scavenging activity.

**Table 3:** Antimicrobial activities oil extracts from garlic bulb and leek leaf oil extracts as mean diameter of zone of inhibition against test pathogenic microbial spp.

Test pathogens	Oil extract	Concentrations of the oil extract (w/v)			Ampicillin (1 *µ*g/ml)
		1 *µ*g/ml	2 *µ*g/ml	3 *µ*g/ml	
*S. boyelli*	Garlic	10.50 ± 0.50^bD^	13.50 ± 0.45^abC^	15.00 ± 0.40^bB^	18.83 ± 0.29^aA^
	Leek	0.00 ± 0.00^cD^	10.50 ± 0.60^cC^	12.93 ± 0.40^cB^	18.50 ± 0.50^aA^

*S. pyogenes*	Garlic	11.00 ± 0.50^abB^	14.03 ± 0.45^aB^	15.50 ± 0.40^abB^	18.83 ± 0.29^aA^
	Leek	11.50 ± 0.45^aD^	13.10 ± 0.36^bC^	16.00 ± 0.50^aB^	18.33 ± 0.29^aA^
					Ketoconazole (1 *µ*g/ml)

*C. albicans*	Garlic	8.50 ± 0.60^aD^	10.50 ± 0.55^aC^	13.50 ± 0.45^aB^	18.00 ± 0.50^aA^
	Leek	0.00 ± 0.00^bD^	10.50 ± 0.40^aC^	12.50 ± 0.50^bB^	17.33 ± 0.76^aA^

*A. niger*	Garlic	0.00 ± 0.00^bD^	8.00 ± 0.95^bC^	11.50 ± 0.50^cB^	17.83 ± 0.28^aA^
	Leek	0.00 ± 0.00^bB^	0.00 ± 0.00^cB^	0.00 ± 0.00^dB^	17.33 ± 0.23^aA^

Means followed by same letter within a column were not significantly different at 0.05 probability level based on the LSD (least significant difference) test. Small letters, significance within column. Capital letters, significance across row. *E. coli*, *Escherichia coli; S. boyelli*, *Shigella boyelli, S. pyogenes*, *Streptococcus pyogenes.*

**Table 4:** MIC, MBC, and MFC of garlic bulb and leek leaf oil extracts.

Test pathogens	Oil extract	MIC (*µ*g/ml)	MBC/MFC (*µ*g/ml)
*S. boyelli*	Garlic	0.50	1.50
	Leek	0.25	0.50

*S. pyogenes*	Garlic	0.40	1.00
	Leek	0.20	0.45

*C. albicans*	Garlic	0.40	0.75
	Leek	0.75	1.25

*A. niger*	Garlic	0.75	1.75
	Leek	0.00	0.00

## Data Availability

The data used to support the findings of this study are included within the supplementary information file.

## Supplementary Materials

Supplementary Materials Table S1. Data for physicochemical properties and antioxidant activities. Table S2. Data for antibacterial activity based on zone of inhibition diameter. Table S3. Data for antifungal activity-based diameter of zone of inhibition.
