# Supplementary material for: Physicochemical Properties and Biological Activities of Garlic (Allium sativum L.) Bulb and Leek (Allium ampeloprasum L. var. Porrum) Leaf Oil Extracts
Source: ScientificWorldJournal. 2022 Apr 26;2022:6573754. doi: 10.1155/2022/6573754 (PMC9064510; doi:10.1155/2022/6573754)
Supplement: Supplementary Materials — Table S1. Data for physicochemical properties and antioxidant activities. Table S2. Data for antibacterial activity based on zone of inhibition diameter. Table S3. Data for antifungal activity-based diameter of zone of inhibition. [file 6573754.f1.pdf]

Table S1. Data for physicochemical properties and antioxidant activities

Oil extract	rep	Oil yield	Specific gravity	Acv	FFA	PV	DPPH	HPSA	AA
Garlic	1	20	0.8	2.805	1.41	4	26.5	11.7	23
Garlic	2	22.5	0.9	2.53	1.27	4.2	28.7	13	27.6
Onion	1	19	0.76	1.4	0.71	2	16.4	10	23.08
Onion	2	16	0.64	1.68	0.85	2.4	18.1	7.9	18.46

Table S2. Data for antibacterial activity based on zone of inhibition diameter

Test pathogens	Oil extract	Rep	Concentrations of the oil extract (w/v)			Ampicillin
			1μg/ml	2μg/ml	3μg/ml	(1μg/ml)
S. boyelli	Garlic	1	10	13	15	19
S. boyelli	Garlic	2	10.5	14	15.5	18.5
S. boyelli	Garlic	3	11	13.5	14.5	19
S. boyelli	Onion	1	0	11	12.5	18
S. boyelli	Onion	2	0	10	13	18.5
S. boyelli	Onion	3	0	10.5	13.3	19
S. pyogenes	Garlic	1	11	14	15.5	19
S. pyogenes	Garlic	2	10.5	14.5	16	18.5
S. pyogenes	Garlic	3	11.5	13.6	15	19
S. pyogenes	Onion	1	12	13	15.5	18.5
S. pyogenes	Onion	2	11	13.5	16	18
S. pyogenes	Onion	3	11.5	12.8	16.5	18.5

Table S3. Data for antifungal activity based diameter of zone of inhibition

Test pathogens	Oil extract	Rep	Concentrations of the oil extract (w/v)			Ketoconazole
			1μg/ml	2μg/ml	3μg/ml	(1μg/ml)
A. versicolor	garlic	1	8	10	14	17.5
A. versicolor	garlic	2	9	11	13	18.5
A. versicolor	garlic	3	8.5	10.5	13.5	18
A. versicolor	onion	1	0	10	12	18
A. versicolor	onion	2	0	10.5	13	17.5
A. versicolor	onion	3	0	11	12.5	16.5
A. niger	garlic	1	0	7	12	18
A. niger	garlic	2	0	8	11	17.5
A. niger	garlic	3	0	9	11.5	18
A. niger	onion	1	0	0	0	17
A. niger	onion	2	0	0	0	17.5
A. niger	onion	3	0	0	0	17.5
